# Efficacy of Roxadustat on anemia and residual renal function in patients new to peritoneal dialysis

**DOI:** 10.1080/0886022X.2022.2050754

**Published:** 2022-03-23

**Authors:** Tong Wu, Yuanyuan Qi, Shuang Ma, Lijie Zhang, Xinyu Pu, Kui Chen, Ying Zhao, Shenghua Sang, Jing Xiao

**Affiliations:** Department of Nephrology, The First Affiliated Hospital of Zhengzhou University, Zhengzhou, China

**Keywords:** Roxadustat, peritoneal dialysis, renal anemia, residual renal function

## Abstract

**Background:**

Both early correction of anemia and preserving residual renal function (RRF) are reported to improve patient survival. The aim of this study was to explore the efficacy and safety of Roxadustat for treatment of renal anemia in patients new to peritoneal dialysis (PD) and to assess its impact on RRF.

**Methods:**

A retrospective analysis was performed on 60 initial peritoneal dialysis (PD) patients with renal anemia. Twenty-eight cases were treated with Roxadustat (Roxadustat group) and 32 with recombinant human erythropoietin (control group). Clinical characteristics, hemoglobin (Hb), C-reactive protein, blood lipids, iron metabolism, dialysis adequacy and RRF of the two groups were evaluated and adverse events were recorded. All patients were followed up for at least 40 weeks.

**Results:**

After 40 weeks of treatment, mean Hb levels were significantly higher from baseline values in both groups, the mean Hb change in Roxadustat group was higher than control group (3.46 ± 1.59 g/dL vs. 2.28 ± 2.27 g/dL, *p* < 0.05). At 40 weeks, 92.9% patients met the target level of Hb in Roxadustat group and 84.4% in control group. Total iron binding was higher and ferritin was lower in Roxadustat group from baseline values and Roxadustat-induced Hb increases were independent of baseline C-reactive protein levels and history of rhuEPO administration. RRF decreased over time in both groups, the mean RRF change was lower in Roxadustat group than control group (1.15 ± 1.66 mL/min/1.73 m^2^ vs. 2.31 ± 1.46 mL/min/1.73 m^2^, *p* < 0.01). Compared with control group, patients in Roxadustat group had higher levels of total iron binding, 24 h urine volume, total weekly Ccr, and lower systolic pressure, ferritin, C-reactive protein, total cholesterol, LDL. No serious adverse reactions occurred in either group.

**Conclusion:**

In patients new to PD, Roxadustat effectively and safely improved renal anemia and delay the decline of RRF.

## Introduction

The prevalence of end-stage renal disease (ESRD) is rising worldwide [1]. Renal anemia, a common complication of ESRD, is associated with lower residual renal function (RRF), increased risk of cardiovascular events, all-cause mortality, and poorer quality of life [2,3]. Studies have demonstrated that the protection of RRF in dialysis patients, especially in new dialysis patients is an independent prognostic factor in patient survival and quality of life [4]. Therefore, it is crucial to achieve Hb target in the early stage of peritoneal dialysis (PD) and protect RRF, thereby improving the long-term prognosis. Recombinant human erythropoietin (rhuEPO) and iron supplements are commonly used to improve hemoglobin levels in PD patients, and the highest doses of rhuEPO are usually required in the early period of PD [5]. Studies have shown that high dosages of ESAs are strongly related to the risk of stroke, cardiovascular disease, cancer and, several clinical problems such as elevated blood pressure, anti-EPO effects, production of anti-EPO antibodies [6,7]. Moreover, only half of PD patients in China treated with rhuEPO have reached Hb levels above 10 g/dL, reasons for the inadequate treatment may include medical costs, inflammation-induced hyporesponsiveness or iron deficiency [8].

Over the past decade, the molecular mechanism of hypoxia-inducible factor (HIF), a transcriptional factor sensing the body’s primary oxygen tension, in regulating erythropoiesis are well understood [9]. Roxadustat (FG-4592), a new oral anti-renal anemia medication, is a HIF prolyl hydroxylase inhibitor inducing HIF stabilization transiently, mimicking the natural erythropoietic response that is associated with transient hypoxia exposure [10]. The intermittent dosing therapy of Roxadustat for treating renal anemia leads to a durable maintenance of the therapeutic effect over time [11]. In recent years, many clinical studies have shown the efficacy and safety of Roxadustat in PD patients with anemia, but study periods are relatively short (≤24 weeks), and study subjects are generally maintenance PD patients, information about efficacy and RRF in new PD patients is limited. Therefore, the present study evaluated the long-term maintenance (40 weeks) effect of Roxadustat on the treatment of anemia and RRF preservation in patients who were on new PD.

## Patients and methods

### Patients

Data of 60 ESRD patients who started PD at the First Affiliated Hospital of Zhengzhou University in China, between November 2019 and October 2020 were included in this study. Inclusion criteria: (1) ages 18–75 years; (2) renal anemia at PD initiation; (3) 24-h urine output ≥ 800 mL before PD initiation; (4) receive maintenance continuous ambulatory peritoneal dialysis (CAPD) and regularly follow up. Exclusion criteria: (1) combine with severe heart failure (NYHA grade 4), severe liver malfunction, malignant tumor, malignant hypertension, autoimmune disease, blood system disease, severe malnutrition before PD initiation; (2) transferred to HD, kidney transplantation, death or lost to follow-up during the whole study period; (3) switch to other anti-anemia drugs; (4) blood transfusion therapy within observational period; (5) history of peritonitis or other severe infection related to PD.

This study was performed in compliance with the Declaration of Helsinki and approved by the Ethics Committee of the First Affiliated Hospital of Zhengzhou University, and was exempted from obtaining informed consents due to the retrospective character (2021-KY-0271).

### Study design

This study was a single-center, retrospective clinical survey. All patients were followed up for at least 40 weeks. Patients were divided into two groups according to the anti-renal anemia drug they were taking at the start of PD: those treated with Roxadustat (*n* = 28) and those treated with rhuEPO (control group, *n* = 32). The Roxadustat group included 20 patients who were changed to Roxadustat from rhuEPO (rhuEPO-Converted subgroup), and 8 patients did not take any anti-anemia drugs before (rhuEPO-naïve subgroup). In accordance with the instructions, the initial dose of Roxadustat was 100 mg (in patients weighing between 45 and <60 kg) or 120 mg (in patients weighing ≥60 kg) three times a week orally, adjusted appropriately in combination with various factors such as basal Hb value, iron metabolism and nutritional status. The follow-up dose was adjusted according to the current Hb level and degree of Hb change over the past 4 week to keep the latter in between 10 and 12 g/dL. Patients in the control group were treated with rhuEPO, with 25 cases previously receiving rhuEPO (rhuEPO-Continued subgroup) and 7 cases not receiving anti-anemia drugs (rhuEPO-Naïve subgroup). The initial dose of rhuEPO was 50–150 IU/kg per week (maximum did not exceed 200 IU/kg per week), administered in one to three subcutaneous injections. The initial treatment goal is an increase in Hb levels of 1–2 g/dL per month, with subsequent adjustments based on the patient’s Hb level, current rhuEPO dose, and treatment response and the target Hb level is 10–12 g/dL. Administration of iron: PD patients with ferritin <100 µg/L and/or transferrin saturation (TSAT) < 20% started oral iron supplementation, the target value of TSAT is 20–50% and ferritin is 100–500 µg/L. Common clinical types of oral iron supplementation include iron polysaccharide complex capsules and iron proteinsuccinylate oral solution.

### Data collection

Baseline demographic data were collected, including gender, age, height, weight, body mass index (BMI), primary kidney disease, complications, comorbidity conditions [a comorbidity score was calculated using the Charlson Comorbidity Index (CCI)] and the medication history. The medication history included ESAs use, iron supplements use, α-blockers, β-blockers, calcium channel blockers (CCB), diuretics, statins and anti-secondary hyperparathyroidism drugs. Various laboratory parameters were recorded throughout the whole study period including systolic pressure, diastolic pressure, red blood cell count (RBC), white blood cell count (WBC), glucose, Hb levels, iron parameters [serum iron, transferrin saturation (TSAT), ferritin, and total iron-binding capacity (TIBC) levels], serum lipids profile [total cholesterol (T-CHO), high-density lipoprotein (HDL) cholesterol, low-density lipoprotein (LDL) cholesterol and triglycerides (TG) levels], serum electrolytes, alanine aminotransferase (ALT), aspartate aminotransferase (AST), serum albumin (Alb), serum C-reactive protein (CRP), serum urea, serum creatinine (Cr), intact parathyroid hormone (iPTH), N-terminal pro-brain natriuretic peptide (NT-proBNP), and 24-h urine volume levels. In addition, baseline dialysis information was collected at the time of stabilized dialysis within four weeks after PD initiation. Dialysis-related indicators included RRF, total weekly Kt/v and total weekly Ccr. RRF were measured in our study using the average of creatinine clearance and urea clearances. The body surface area was calculated based on the Gehan and George equation [12].

$$\mathrm{RRF}=\frac{1}{2}[\frac{\mathrm{UrineCr}(\mu \mathrm{mol}/L)}{\mathrm{SerumCr}(\mu \mathrm{mol}/L)}+\frac{\mathrm{UrineUrea}(\mathrm{mmol}/L)}{\mathrm{SerumUrea}(\mathrm{mmol}/L)}]\times \frac{\text{Urine Volume}(\mathrm{mL})}{1440}$$

### Statistical analysis

Continuous variables in accordance with normal distribution were expressed as mean ± standard deviation (SD) and data not conforming to normal distribution were presented as the median and interquartile range. Categorical variables were described as numbers and percentages. Comparisons of the measurements between two groups were performed using the independent t-test for normally-distributed data and the Mann-Whitney *U*-test for non-normally-distributed data. Comparisons of categorical variables between two groups were performed using chi-square test or Fisher’s exact-test. Changes in each group during treatment were analyzed using the paired t-test for normally-distributed data and the Wilcoxon signed rank test for non-normally-distributed data. Repeated-measures analysis of variance was used to further assess the effect of group differences on longitudinal changes in RRF. Gender and baseline CCI score were included as covariates to adjust the analysis. The values of ferritin and BNP levels were not normally distributed. So, they were log-transformed before performing the above parametric analysis. *p*-Value <0.05 was considered statistically significant in all statistical analyses. All statistical analyses were conducted using SPSS version 25.0 (IBM Corp, Armonk, NY).

## Results

### Demographic and clinical characteristics

**Figure 1.**
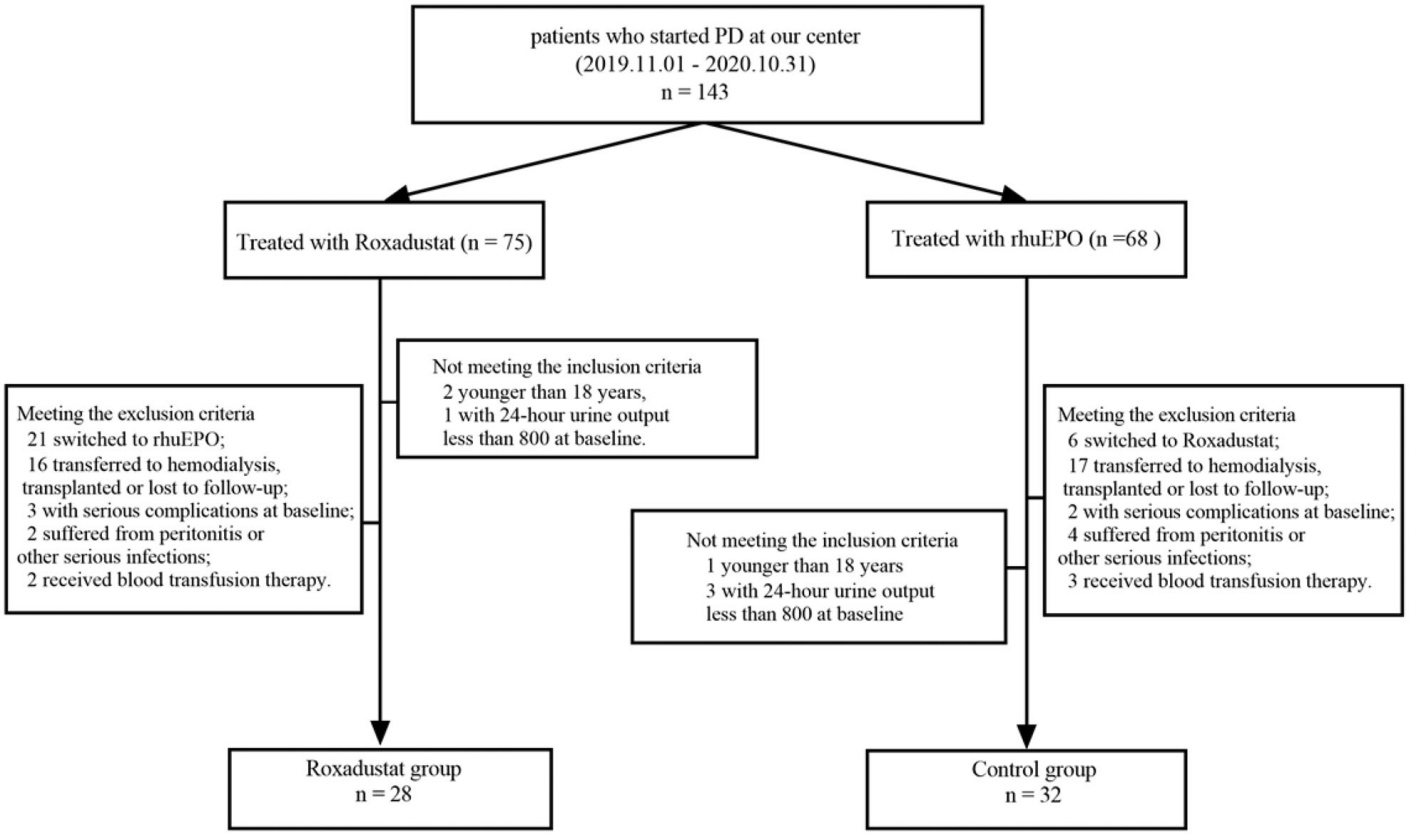
Flow chart of patient screening. PD: peritoneal dialysis.

A total of 60 patients were enrolled in this study, with 28 in the Roxadustat group and 32 in the control group. The flow chart for patient screening is shown in Figure 1. At the start of the study, 20 patients in the Roxadustat group were switched from rhuEPO to Roxadustat, for reasons of low rhuEPO responsiveness (*n* = 6) and low compliance (*n* = 14); 22 patients in the control group received rhuEPO medication before PD. The mean dose of previous rhuEPO and Hb levels between the two groups were shown in Table 1. There were no significant differences in previous rhuEPO doses and baseline Hb levels between the two groups, nor were the differences in the mean Hb levels at baseline statistically significant within the Roxadustat and control subgroups (shown in Table 1).

**Table 1.** Mean dose of previous rhuEPO and Hb levels between the two groups.

	Roxadustat group		Control group	
	rhuEPO-Converted group (20)	rhuEPO-Naïve group (8)	rhuEPO-Continued group (25)	rhuEPO-Naïve group(7)
Vintage (month)	12.04 ± 2.95	13.18 ± 3.04	12.5 ± 2.50	12.96 ± 2.32
rhuEPO dose at baseline (IU/kg/week)	202.69 ± 74.72	0	162.03 ± 91.99	0
Hb at baseline(g/dL)	8.19 ± 1.38	8.00 ± 1.11	8.80 ± 1.42	7.61 ± 1.31

rhuEPO-Converted subgroup: patients previously receiving rhuEPO; rhuEPO-naïve subgroup: not previously receiving rhuEPO; rhuEPO-Continued: patients continuously receiving rhuEPO.

Table 2 showed the demographic and laboratory data of the Roxadustat and control group at baseline. Among these patients, the average age was 50.4 ± 11.9 years, and 58.3% were male. Results showed that 83.3% had hypertension, 16.7% had cardiovascular disease (CVD), and 8.3% had diabetes before PD initiation. Primary causes of ESRD in these patients were as follows: chronic glomerulonephritis (71.7%), hypertensive nephrosclerosis (13.3%), diabetic nephropathy (3.3%), others or unknown (11.7%). There were no significant differences in the baseline clinical characteristics, major comorbid conditions, laboratory findings, dialysis adequacy indices and RRF between the two groups.

**Table 2.** Demographic and baseline laboratory data between the two groups.

Characteristic	Roxadustat group (*n* = 28)	Control group (*n* = 32)	*p*
Age (years)	39.71 ± 9.61	43.44 ± 11.89	0.192
Men [*n* (%)]	17 (60.7)	18 (56.3)	0.726
BMI (Kg/m^2^)	23.79 ± 4.23	23.52 ± 3.56	0.791
Systolic blood pressure (mmHg)	133.68 ± 12.51	134.53 ± 14.96	0.813
Diastolic blood pressure (mmHg)	83.39 ± 8.97	82.00 ± 7.78	0.522
Major comorbidity [*n* (%)]			
Hypertension	24 (85.7)	26 (81.3)	0.643
Diabetes	3 (10.7)	2 (6.3)	0.657
Cardiovascular disease	4 (14.3)	6 (18.8)	0.643
Cerebrovascular disease	2 (7.1)	3 (9.4)	1.000
Primary kidney disease [*n* (%)]			
Glomerulonephritis	21 (75.0)	22 (68.8)	0.592
Hypertensive nephrosclerosis	3 (10.7)	5 (15.6)	0.712
Diabetic nephropathy	1 (3.6)	1 (3.1)	1.000
Others (or unknown)	3 (10.7)	4 (12.5)	1.000
Charlson comorbidity index score	3.36 ± 1.25	3.56 ± 1.16	0.513
White blood cell count (10^9^/L)	5.17 ± 1.27	5.71 ± 1.91	0.209
Red blood cell count (10^12^/L)	2.68 ± 0.46	2.82 ± 0.45	0.228
Hemoglobin (g/dL)	8.13 ± 1.29	8.54 ± 1.47	0.264
Serum iron (µmol/L)	14.37 ± 4.83	14.00 ± 3.57	0.736
Log_10_Ferritin (ng/mL)	2.33 ± 0.49	2.26 ± 0.43	0.601
TIBC (µmol/L)	43.26 ± 7.74	43.21 ± 6.13	0.977
TSAT (%)	0.34 ± 0.11	0.33 ± 0.10	0.822
K (mmol/L)	4.58 ± 0.58	4.73 ± 0.72	0.381
NA (mmol/L)	138.42 ± 2.47	138.70 ± 3.81	0.729
CL (mmol/L)	104.11 ± 5.63	102.67 ± 4.75	0.285
Ca (mmol/L)	1.98 ± 0.23	2.08 ± 0.24	0.107
P (mmol/L)	2.08 ± 0.62	2.10 ± 0.46	0.862
iPTH (pmol/L)	299.55 ± 121.49	262.55 ± 139.22	0.280
BUN (µmol/L)	36.31 ± 8.85	36.23 ± 8.11	0.970
Serum Cr (µmol/L)	980.27 ± 239.49	960.20 ± 213.67	0.733
Uric acid (µmol/L)	453.14 ± 140.02	425.19 ± 138.81	0.441
ALT (U/L)	13.79 ± 7.68	14.94 ± 9.40	0.609
AST (U/L)	14.25 ± 4.01	16.50 ± 6.33	0.102
Serum albumin (g/L)	39.68 ± 7.84	37.55 ± 4.17	0.269
Total cholesterol (mmol/L)	3.67 ± 0.90	3.89 ± 1.22	0.418
Triglyceride (mmol/L)	1.18 ± 0.58	1.31 ± 0.48	0.360
HDL cholesterol (mmol/L)	0.97 ± 0.31	0.98 ± 0.29	0.896
LDL cholesterol (mmol/L)	2.24 ± 0.74	2.38 ± 0.85	0.503
C-reactive protein (mg/L)	1.90 (0.83 ∼ 4.50)	2.30 (0.78 ∼ 3.43)	0.683
LVEF (%)	61.75 ± 2.91	61.81 ± 3.10	0.659
Log_10_ NT pro-BNP (pg/mL)	3.40 ± 0.47	3.39 ± 0.53	0.945
Urine volume (mL/24h)	1681.43 ± 428.76	1529.69 ± 546.08	0.241
Total weekly Kt/V	1.77 ± 0.43	1.82 ± 0.35	0.629
Renal weekly Kt/V	0.80 ± 0.31	0.84 ± 0.30	0.680
Peritoneal weekly Kt/V	0.94 ± 0.30	0.99 ± 0.33	0.547
Total weekly Ccr	74.91 ± 19.93	71.97 ± 20.08	0.573
Peritoneal weekly Ccr	30.82 ± 6.99	31.62 ± 8.33	0.694
Renal weekly Ccr	44.08 ± 19.80	40.35 ± 19.60	0.468
RRF (ml/min/1.73 m^2^)	4.32 ± 1.65	4.12 ± 1.69	0.644
Medication [*n* (%)]			
rhuEPO	20 (71.4)	25 (78.1)	0.550
CCB	25 (89.3)	30 (93.8)	0.657
Diuretics	2 (7.1)	2 (6.3)	1.000
α-blockers	15 (53.6)	13 (40.6)	0.316
Β-blockers	13 (46.4)	15 (46.9)	0.972
Statins	6 (21.4)	3 (9.4)	0.192
Anti-secondary hyperparathyroidism drugs	21 (75.0)	19 (59.4)	0.200

BMI: body mass index; TIBC: total iron-binding capacity; TSAT: transferrin saturation; iPTH: intact parathyroid hormone; BUN: blood urea nitrogen; ALT: alanine aminotransferase; AST: aspartate aminotransferase; HDL: High-Density Lipoprotein; LDL: Low-Density Lipoprotein; LVEF: left ventricular ejection fraction; NT pro-BNP: N-terminal pro-brain natriuretic peptide; Ccr: creatinine clearance; Kt/V: urea clearance; RRF: residual renal function; rhuEPO: recombinant human erythropoietin; CCB: calcium channel blocker. *p* < 0.05 was defined as statistically significant.

### Effects of Roxadustat on Hb

**Figure 2.**
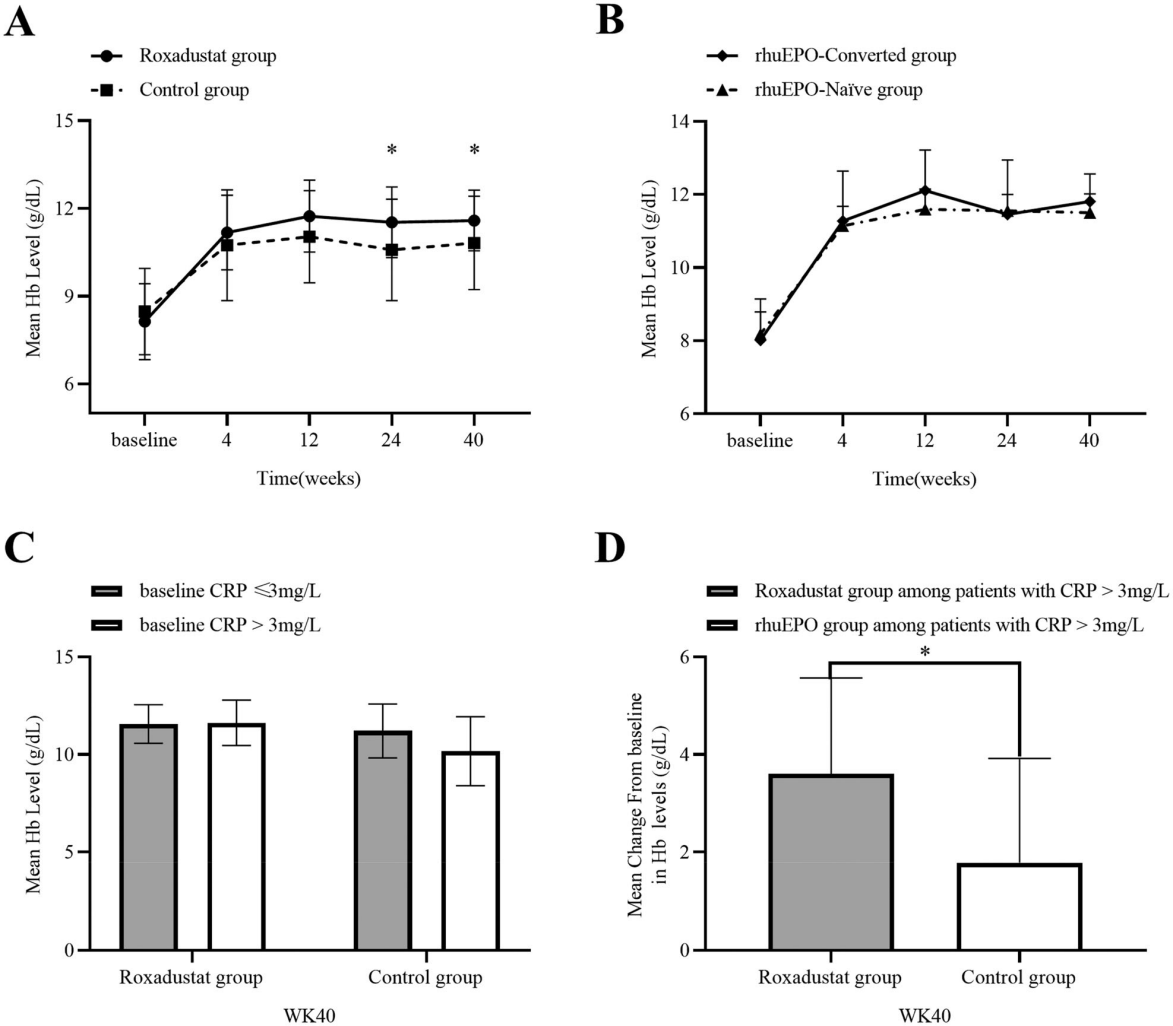
(A) Changes in Hb levels over time in the Roxadustat group and rhuEPO group. (B) Changes in hemoglobin levels over time in the rhuEPO-Converted group and rhuEPO-naïve group. (C) Mean hemoglobin levels at 40 weeks in the Roxadustat group and rhuEPO group according to the CRP subgroup. (D) Mean change from baseline in Hb levels among patients with higher CRP levels at week 40. Values are expressed as means ± SD. *p < 0.05 (A) vs. the Control group. *p < 0.05 (D) vs. the rhuEPO subgroup among patients with CRP > 3 mg/L.

At baseline, mean Hb level was 8.13 ± 1.29 g/dL in the Roxadustat group and 8.54 ± 1.47 g/dL in the control group. At 40 weeks, mean value was 11.59 ± 1.04 g/dL and 10.82 ± 1.59 g/dL, respectively. The mean change from baseline in Hb was significantly higher in the Roxadustat group than the control group at 24 weeks (3.40 ± 1.74 g/dL vs.2.05 ± 2.49 g/dL, *p* < 0.05) and 40 weeks (3.46 ± 1.59 g/dL vs. 2.28 ± 2.27 g/dL, *p* < 0.05). The inter-group difference was significant at 24 and 40 weeks (both *p* < 0.05) (shown in Figure 2(A)). The percentage of patients reaching target Hb level (10–12 g/dL) was 92.9% (*n* = 26) in the Roxadustat group and 84.4% (*n* = 27) in the control group at 40 weeks. In the Roxadustat group, a significant increase of Hb levels was observed in both rhuEPO-Converted subgroup and rhuEPO-Naïve subgroup during the study period. There was no significant difference in Hb level between two subgroups at each time point (shown in Figure 2(B)).

Patients were further divided into CRP > 3 mg/L and CRP ≤ 3 mg/L subgroups based on baseline CRP levels. At the end of the study, patients with high CRP levels (*n* = 11) and patients with low CRP levels (*n* = 17) had similar Hb levels (11.57 ± 0.99 g/dL vs. 11.63 ± 1.16 g/dL, *p* = 0.888) in the Roxadustat group, while in the control group, patients with high CRP levels (*n* = 12) had slightly lower mean Hb levels than those with low CRP levels (*n* = 20) (11.21 ± 1.38 g/dL vs.10.17 ± 1.77 g/dL, *p* = 0.074) (shown in Figure 2(C)). Moreover, among patients with high CRP levels, the mean Hb level change from baseline was greater in the Roxadustat group than in the control group (shown in Figure 2(D)).

### Changes in doses of Roxadustat and rhuEPO

**Figure 3.**
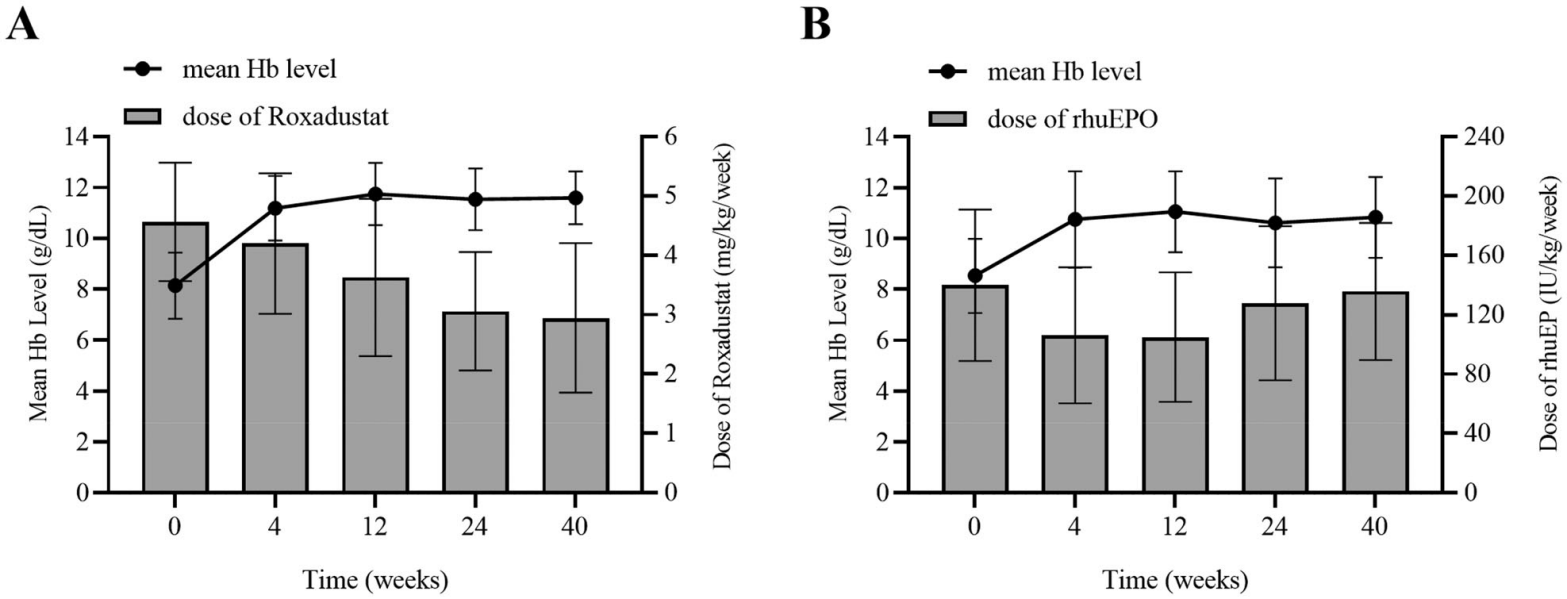
(A) Changes in doses and Hb levels over time in the Roxadustat group. (B) Changes in doses and Hb levels over time in the rhuEPO group.

During treatment, the dose of Roxadustat decreased from 4.56 ± 1.00 mg/kg/week at baseline to 3.06 ± 0.99 mg/kg/week at 24 weeks, with no significant difference in dose at 40 weeks versus 24 weeks (2.94 ± 1.26 mg/kg/week vs. 3.06 ± 0.99 mg/kg/week, *p* > 0.05). In the control group, the doses of rhuEPO decreased from 175.56 ± 95.83 IU/kg/week at baseline to 112.51 ± 56.77 IU/kg/week at 12 weeks and then gradually increased to 148.47 ± 61.97 IU/kg/week at 40 weeks. The mean Hb levels increased significantly from baseline in both groups and were maintained at high levels (≥10 g/L) (shown in Figure 3).

### Effects of Roxadustat on iron metabolism

**Figure 4.**
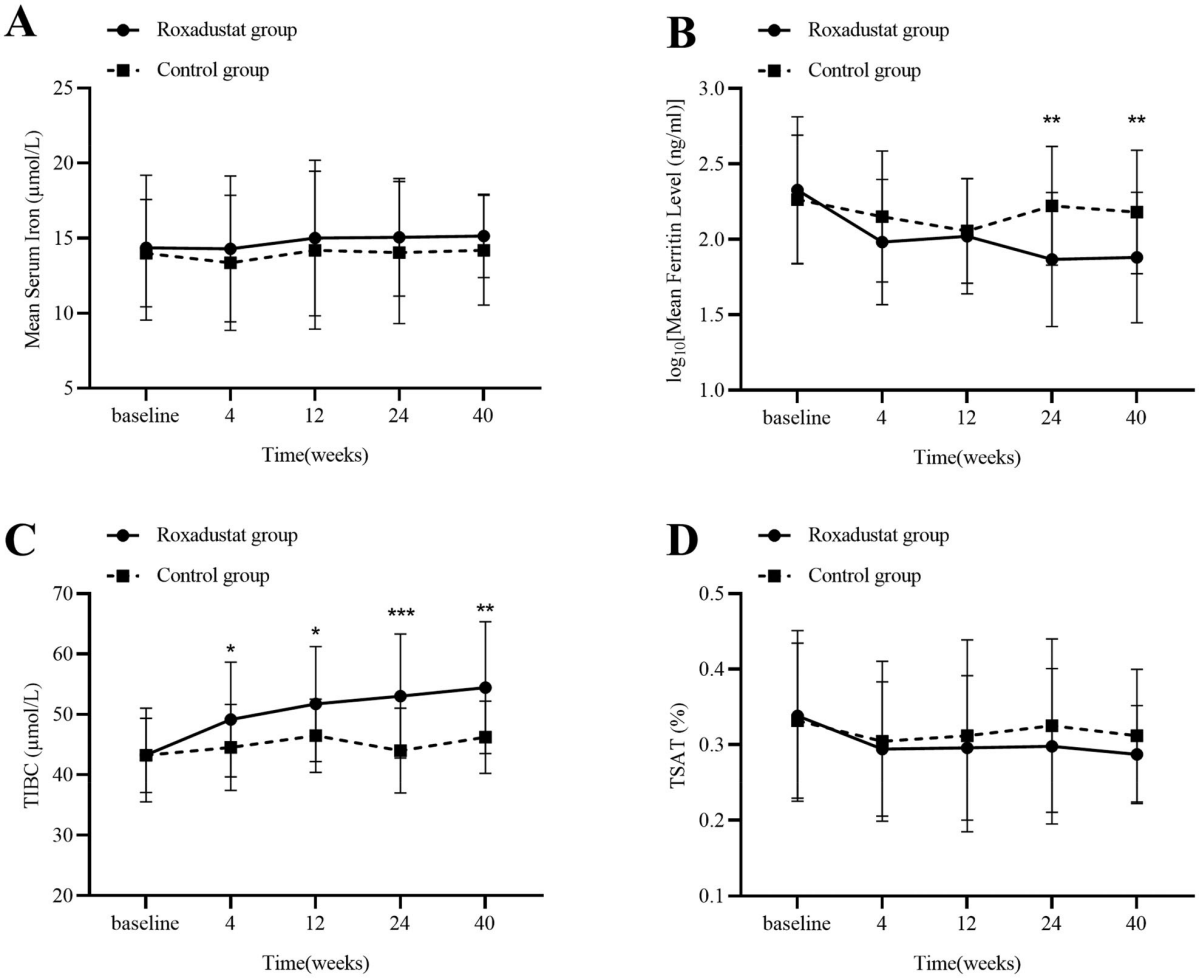
Changes in serum iron level (A), logarithm of serum ferritin level (B), TIBC level (C) and TSAT level (D) over time in the Roxadustat group and Control group. Values are expressed as means ± SD. **p < 0.01 (B) vs. the Control group.*p < 0.05, **p < 0.01,***p < 0.001(C) vs. the Control group. TIBC: total iron-binding capacity; TSAT: transferrin saturation.

During the follow-up period, nine patients (32.1%) in the Roxadustat group received oral iron therapy, as compared with 26 (81.3%) in the control group. No significant changes in serum iron levels were observed in both groups (shown in Figure 4(A)). Compared with baseline values, Roxadustat group showed a significant increase in TIBC and a significant decrease in ferritin at each time point; the control group showed a significant increase in TIBC and a decrease in ferritin at 12 weeks, without significant differences at the other time points. The change from baseline in TIBC was higher in the Roxadustat group than in the rhuEPO group at each time point of treatment (all *p* < 0.05), and the change values were 11.17 ± 11.12 µmol/L and 3.00 ± 8.87 µmol/L at 40 weeks respectively (shown in Figure 4(B,C)). In addition, there was a significant decrease in TSAT levels in Roxadustat group from the baseline values, while no significant change was observed in the control group (shown in Figure 4(D)).

### Effects of Roxadustat on RRF

**Figure 5.**
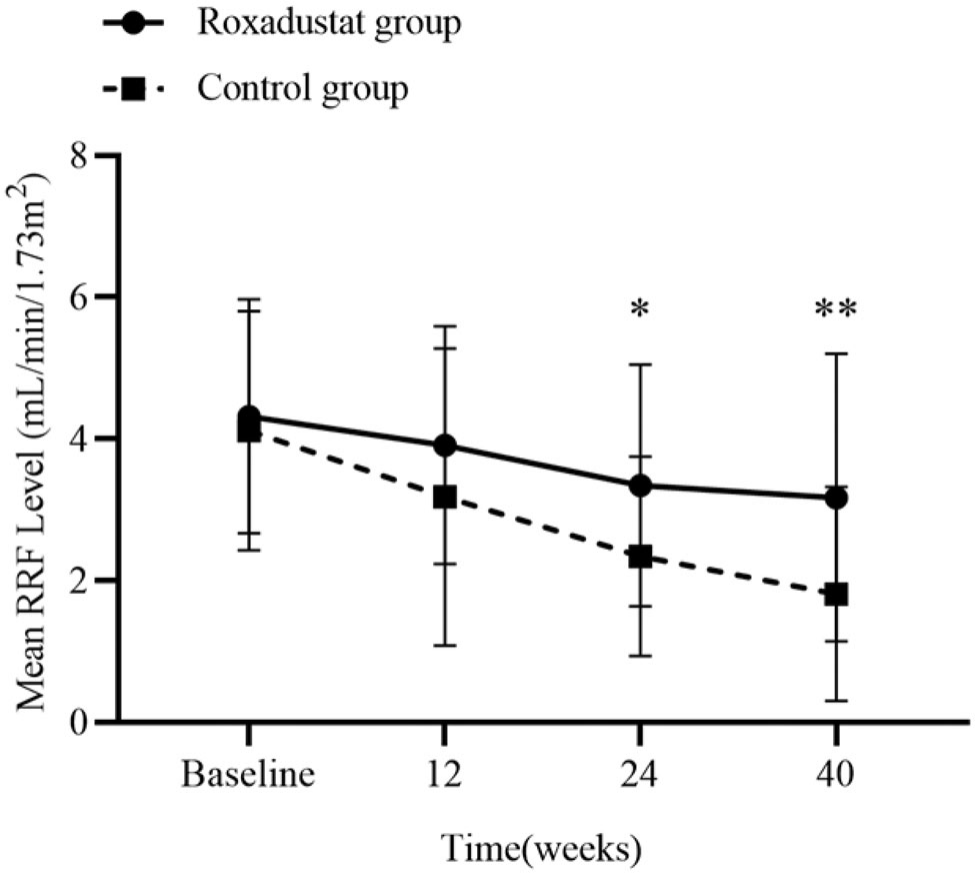
Residual renal function at baseline and during follow-up in the two groups. RRF, residual renal function.

RRF gradually decreased after PD initiation in both groups. RRF was 4.32 ± 1.65 mL/min/1.73 m^2^ at baseline, 3.91 ± 1.67 mL/min/1.73 m^2^ at 12 weeks (*p* > 0.05), 3.34 ± 1.71 mL/min/1.73 m^2^ at 24 weeks (*p* < 0.001) and 3.17 ± 2.03 mL/min/1.73 m^2^ at 40 weeks (*p* < 0.001) in the Roxadustat group. In the control group, RRF was 4.12 ± 1.69 mL/min/1.73 m^2^ at baseline, 3.18 ± 2.10 mL/min/1.73 m^2^ at 12 weeks (*p* < 0.001), 2.34 ± 1.41 mL/min/1.73 m^2^ at 24 weeks (*p* < 0.001) and 1.81 ± 1.51 mL/min/1.73 m^2^ at 40 weeks (*p* < 0.001). The mean level of decline in RRF from baseline was lower in the Roxadustat group than the control group at 24 weeks (0.98 ± 1.28 mL/min/1.73 m^2^ vs. 1.78 ± 1.45 mL/min/1.73 m^2,^ *p* < 0.05) and 40 weeks (1.15 ± 1.66 vs. 2.31 ± 1.46, *p* < 0.01). The RRF level was significantly higher in Roxadustat group than in the control group at 24 weeks and 40 weeks (shown in Figure 5). Based on repeated-measures analysis of variance, there were significant changes in RRF over time (*F* = 3.930, *p* < 0.001), and there was a significant difference in the effect of grouping factor on RRF (*F* = 4.429, *p* = 0.044). There was no interaction between the time factor and grouping factor (*F* = 2.959, *p* = 0.052). Furthermore, gender and baseline CCI score were included as covariates to adjust the analysis, time factor and grouping factor presented significant effects on RRF change (both *p* < 0.05) but not on the gender and CCI score (both *p* > 0.05). The interaction between time and grouping factor was significant (*F* = 2.827, *p* = 0.047).

### Effects of Roxadustat on dialysis-related indicators

**Figure 6.**
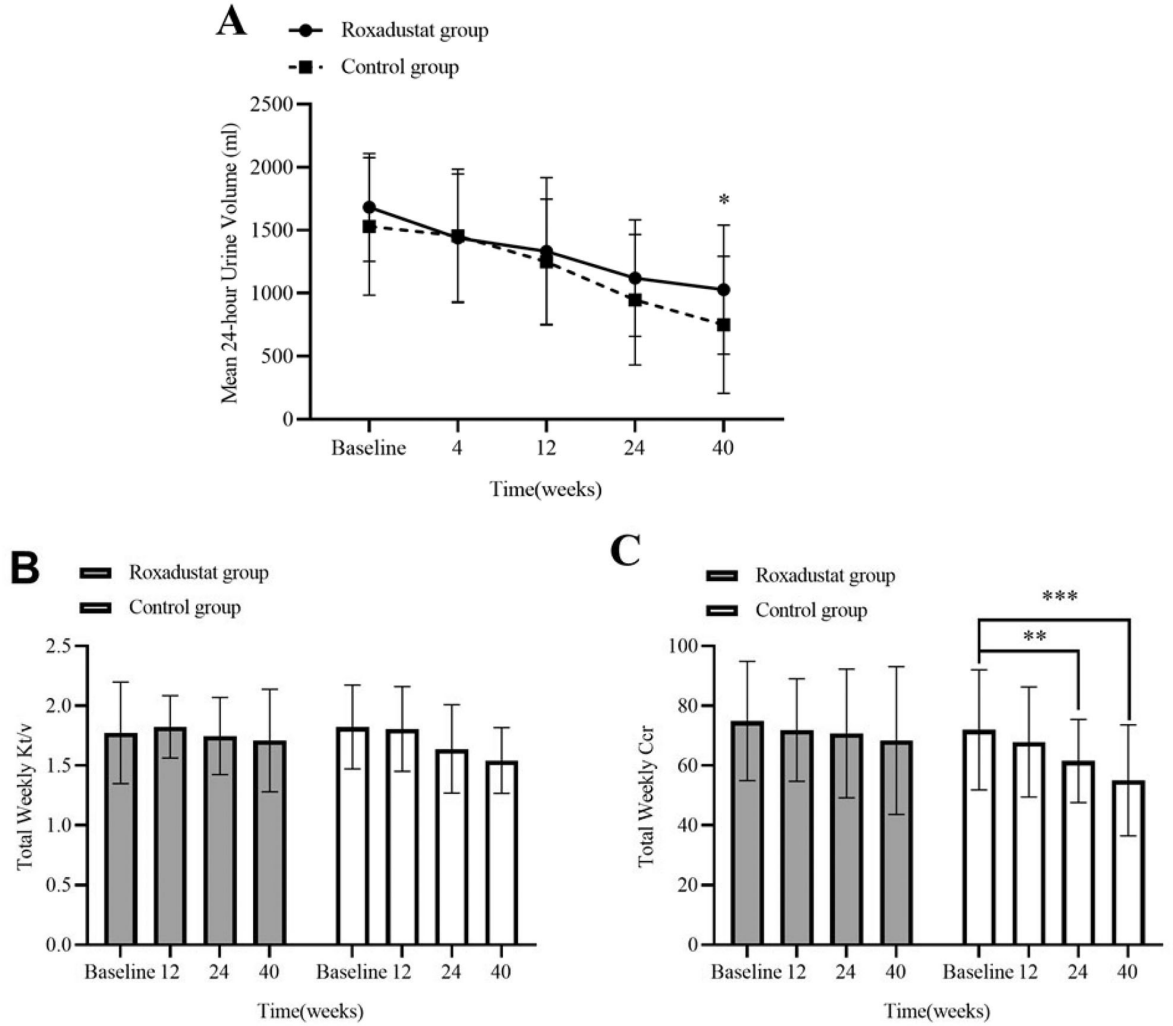
Changes in 24-h urine volume (A), Total weekly Kt/v (B), and Total weekly Ccr (C) during 40 weeks after PD initiation in the Roxadustat group and ESAs group. Values are expressed as means ± SD. *p < 0.05 vs. baseline level. **p < 0.01 vs. baseline level. ***p < 0.001 vs. baseline level.

A decrease in 24-h urine volume over time was observed in either group during the study. At 40 weeks, the change from baseline in 24-h urine volume was 652.50 ± 645.24 mL in the roxadustat group and 781.09 ± 724.62 mL in the control group. There was a significant difference in 24-h urine volume between the two groups at 40 weeks (1028.90 ± 512.72 mL vs. 748.59 ± 543.10 mL, *p* < 0.05) (shown in Figure 6(A)). Total weekly Kt/V levels did not significantly change during the study period in either group, although it tended to decrease in the control group (shown in Figure 6(B)). Total weekly Ccr levels showed a slight decrease that was not statistically different from the baseline value in the roxadustat group, and the control group showed significant differences in total weekly Ccr levels from baseline values at 24 weeks (*p* < 0.01) and 40 weeks (*p* < 0.001) (shown in Figure 6(C)). Moreover, there was a significant difference in total weekly Ccr between the two groups at 40 weeks (68.38 ± 24.75 vs. 55.04 ± 18.56, *p* < 0.05).

### Changes in other clinical indices between the two groups during the study

Table 3 showed the difference in other clinical indices between the two groups. The mean total cholesterol and LDL cholesterol levels were significantly lower in the Roxadustat group than in the control group at 12 weeks and 40 weeks. Moreover, patients who were treated with Roxadustat showed lower levels of systolic blood pressure, and C-reactive protein (CRP) at the end of the study compared with rhuEPO.

**Table 3.** Comparison of other clinical indices between the two groups.

	After 12 weeks		*p*	After 24 weeks		*p*	After 40 weeks		*p*
	Roxadustat group (*n* = 28)	Control group (*n* = 32)		Roxadustat group (*n* = 28)	Control group (*n* = 32)		Roxadustat group (*n* = 28)	Control group (*n* = 32)	
Systolic BP (mmHg)	136.39 ± 11.59	139.19 ± 9.94	0.319	133.93 ± 11.45	138.03 ± 11.28	0.168	134.36 ± 13.90	140.81 ± 10.58	0.046
Diastolic BP (mmHg)	82.46 ± 7.38	84.47 ± 6.37	0.263	84.25 ± 6.50	84.28 ± 8.77	0.988	83.14 ± 6.60	84.75 ± 6.74	0.356
Total cholesterol (mmol/L)	3.41 ± 0.93	3.95 ± 0.82	0.018	3.35 ± 0.64	3.67 ± 0.79	0.098	3.41 ± 0.67	3.76 ± 0.65	0.042
Triglyceride (mmol/L)	1.15 ± 0.94	1.36 ± 0.62	0.294	1.09 ± 0.67	1.27 ± 0.79	0.290	1.18 ± 0.97	1.26 ± 0.53	0.678
HDL cholesterol (mmol/L)	0.97 ± 0.26	0.99 ± 0.26	0.702	0.97 ± 0.22	1.02 ± 0.28	0.451	1.01 ± 0.27	1.05 ± 0.28	0.628
LDL cholesterol (mmol/L)	2.01 ± 0.65	2.33 ± 0.46	0.029	2.02 ± 0.53	2.24 ± 0.50	0.110	1.96 ± 0.47	2.27 ± 0.59	0.032
iPTH (pmol/L)	173.41 ± 124.81	184.91 ± 120.46	0.718	228.51 ± 210.19	217.31 ± 113.84	0.804	217.19 ± 127.24	240.65 ± 156.06	0.530
Serum albumin (g/L)	33.65 ± 4.76	33.89 ± 5.16	0.851	33.15 ± 3.51	34.35 ± 4.17	0.238	33.25 ± 2.86	33.83 ± 3.62	0.486
K (mmol/L)	4.10 ± 0.51	4.39 ± 0.72	0.078	4.07 ± 0.68	4.36 ± 0.62	0.083	4.20 ± 0.68	4.39 ± 0.57	0.252
C-reactive protein (mg/L)	1.35 (0.53 ∼ 3.45)	1.85 (0.70 ∼ 3.60)	0.722	1.05 (0.08 ∼ 3.09)	2.10 (0.70 ∼ 5.03)	0.181	1.10 (0.50 ∼ 3.26)	2.60 (1.10 ∼ 5.38)	0.042
Log_10_ (NT pro-BNP) (pg/ml)	3.28 ± 0.54	3.31 ± 0.56	0.841	3.29 ± 0.56	3.51 ± 0.54	0.123	3.33 ± 0.64	3.62 ± 0.61	0.079
Peritoneal weekly Ccr	32.49 ± 9.56	34.00 ± 8.68	0.524	33.89 ± 7.63	35.74 ± 8.25	0.372	36.76 ± 10.74	36.86 ± 8.99	0.971
Renal weekly Ccr	39.40 ± 19.67	33.91 ± 17.09	0.252	36.86 ± 22.82	25.79 ± 14.65	0.027	31.61 ± 27.76	18.19 ± 18.39	0.022

BP: blood pressure; HDL: High-Density Lipoprotein; LDL: Low-Density Lipoprotein; iPTH: intact parathyroid hormone; NT pro-BNP: N-terminal pro-brain natriuretic peptide; Ccr: creatinine clearance; Kt/V: urea clearance. *p* < 0.05 was defined as statistically significant.

### Cardiovascular safety and adverse events

During the study period, there were no patients with major cardiovascular events (MACE) in the Roxadustat group. One patient in the control group hospitalized because of heart failure. But cardiac function was improved after treatment. Table 4 showed the incidence of adverse events between the two groups. The number of patients with Hb levels above the target value(>12g/dL) was nine in the Roxadustat group and six in the control group. There was no significant difference in the incidence of adverse reactions between the two groups.

**Table 4.** The incidence of adverse events between the two groups.

	Roxadustat group (*n* = 28))	Control group (*n* = 32)	*p*
Hb level above target *n* (%)	9 (32.1)	6 (18.8)	0.232
Hypertension *n* (%)	4 (14.30)	11 (34.40)	0.073
Hyperkalemia *n* (%)	4 (14.30)	3 (9.40)	0.695
Nausea *n* (%)	2 (7.1)	3 (9.40)	1.000
Diarrhea *n* (%)	1 (3.60)	0 (0.00)	0.467
Dizziness *n* (%)	2 (7.1)	4 (12.5)	0.675
Abnormal liver function *n* (%)	0(0.00)	1(3.10)	1.000

## Discussion

Initiating PD treatment brings new life to ESRD patients. However, patients shortly after starting dialysis represent a vulnerable group and almost all of them have varying degrees of renal anemia, the severer the anemia, the higher the risk of death, cardiovascular events and hospitalization [2]. In the present study, patients in the Roxadustat group showed an increase in mean Hb levels of 3.46 g/dL from baseline values, with an Hb target rate of 92.9% after 40 weeks of treatment, suggesting that Roxadustat effectively improved Hb levels in patients who were on new PD and maintained the Hb target rate at a high level after long-term administration. Our results of subgroup analysis based on previous treatment with rhuEPO or not and baseline CRP levels showed that neither baseline CRP nor history of rhuEPO administration affected the efficacy of Roxadustat with respect to Hb levels, which were consistent with the reports on PD patients by Hou et al. [13]. Changes in dose of the two drugs showed that the mean dose of Roxadustat stabilized after a gradual decrease, maintaining Hb at 11.59 g/dL with a maintenance dose of 2.94 mg/kg/week, while for rhuEPO the mean dose gradually increased after a short-term decrease, with a mean dose of 148.47 IU/kg/week and Hb of 10.82 g/dL at the end of the study, indicating that Roxadustat could maintain a higher Hb level with a more stable dose change than rhuEPO. Additionally, in contrast to a phase III clinical trial aimed at PD patients in Japan [14], the initial and maintenance dose of Roxadustat in our study is slightly higher than that in the Japanese study, we analyze that the reasons for dose differences may be related to the baseline Hb value of the study subjects and drug responsiveness.

With regard to iron metabolism, patients in the rhuEPO group showed only a transient increase in TIBC and a decrease in ferritin at 12 weeks, which may be associated with the early rapid increase in Hb synthesis. In the Roxadustat group, only nine patients took oral iron supplementation, TIBC increased from baseline values and ferritin decreased at all time points, while serum iron remained stable during the study, reflecting the fact that Roxadustat mobilizes the body's iron reserves, increases iron transport capacity and improves iron utilization, ensuring Hb synthesis even in the absence of routine iron supplementation in PD patients [15].

RRF is of great significance for the survival of PD patients, and a rapid decline in RRF is a strong predictor of cardiovascular as well as all-cause mortality in PD patients [16]. An extra important issue of this research was the change in RRF in the Roxadustat group, which showed a better RRF compared with rhuEPO group. After adjusting covariates, the effect remained. However, the effect of Roxadustat on RRF was not found in the study by Hirai et al. [17]. The reasons for the different results may be related to the the target subjects and duration of follow-up. The study by Hirai was conducted in patients undergoing maintenance PD with a follow-up period of 24 weeks.

The reasons for the protective effect of Roxadustat on RRF may be multifactorial. Micro-inflammatory states are prevalent in PD patients and closely associated with the decline of RRF [18]. CRP is a well-recognized marker for local–systemic inflammation [19]. The result of our study showed that after 40 weeks of treatment, CRP levels in the Roxadustat group were significantly lower than in the rhuEPO group, which may be associated with the fact that Roxadustat can promote the expression of adenosine genes through HIF and assist adenosine to exert anti-inflammatory effects [20]. The potential anti-inflammatory effect of Roxadustat was also recently identified in a study by Yang et al. [21]. Furthermore, inflammation interferes with the body’s iron homeostasis through hepcidin and cause functional iron deficiency, which in turn increase EPO resistance [22]. Both EPO resistance and low Hb level accelerate the decline of RRF [23]. Many clinical trials have shown that Roxadustat can significantly reduce hepcidin and can potentially be used for the treatment of inflammation-induced anemia in PD patients [13,14]. The results of subgroup analysis based on baseline hsCRP levels showed that 83.3% of patients with high CRP levels in the rhuEPO group had rhuEPO doses >10,000 IU/week, but patients with high CRP levels had a slightly lower Hb response than patients with low CRP levels, and among patients with high CRP levels, Roxadustat showed a greater Hb response than those to rhuEPO, It suggested that inflammation might inhibit the rhuEPO response, whereas the Hb response of Roxadustat is independent of the inflammatory microenvironment, which is consistent with previous reports in PD non-dialysis and dialysis patients with renal anemia [5,24]. Therefore, the protective effect of Roxadustat on RRF may be related to the control of microinflammation and improvement of inflammatory anemia.

The protective effect of Roxadustat on RRF may also be partly derived from improving the body's lipid metabolism. Combined disorders of lipid metabolism in PD patients can lead to further glomerular damage through renal atherosclerotic plaque formation, lipid-induced oxidative stress or promoting the release of inflammatory cytokines, resulting in a rapid decline in RRF [25]. Studies have shown that Roxadustat can have a cholesterol-lowering effect by promoting the degradation of 3-hydroxy-3-methylglutaryl coenzyme A reductase (a key enzyme in cholesterol synthesis) through HIF [26]. Lower mean T-CHO and LDL levels were also found in patients with Roxadustat than rhuEPO in our study. Therefore, it is hypothesized that Roxadustat has potential lipid-lowering effects and helps to prevent atherosclerosis and delay the decline of RRF. Additionally, a higher total weekly CCr was found in the Roxadustat group than the control group, while there was no significant difference in peritoneal weekly CCr between the two groups. This effect appears to be mostly a result of maintaining RRF.

Data from several previous studies suggest that high dose of rhuEPO increase the risk of high blood pressure, cardiovascular events and mortality [27,28]. The overall trend of rhuEPO dose changes in this study was upward, reaching 148.47 IU/kg/week at 40 week, which may lead to the higher mean blood pressure level in the rhuEPO group than Roxadustat group. A latest animal experiment showed that Roxadustat can alleviate hypertension and organ damage through upregulation of angiotensin receptor type 2 (AGTR2) and endothelial NO synthase (eNOS), downregulation of angiotensin receptor type 1 (AGTR1), and inhibition of oxidative stress [29]. No significant change from baseline in blood pressure levels in the Roxadustat group was found in our study, and further studies are needed to explore whether Roxadustat has an antihypertensive effect in clinical practice.

Some limitations need to be clarified. First, this was a retrospective observational study and selection bias could not be completely eliminated. Second, we selected patients who were on CAPD modality and did not suffer from serious peritonitis as the study subjects during the follow-up period. Gender and baseline CCI score were introduced to the analysis as covariates. However, other potential confounding factors that influenced RRF could not be completely excluded. Finally, the follow-up period was relatively short and the sample size was small. Therefore, the effects of Roxadustat on RRF in newly PD patients presented here should be regarded as preliminary, further prospective, large randomize clinical trials are needed to testify the finding.

## Conclusion

The present study showed that Roxadustat effectively improved hemoglobin concentration, maintaining a high target rate of Hb during treatment, and delay the decline in RRF in patients new to PD.

## Data Availability

The datasets used and/or analyzed in this study are available from the corresponding author on reasonable request.
